# Osthole: An up-to-date review of its anticancer potential and mechanisms of action

**DOI:** 10.3389/fphar.2022.945627

**Published:** 2022-09-07

**Authors:** Shaojie Yang, Wanlin Dai, Jingnan Wang, Xiaolin Zhang, Yuting Zheng, Shiyuan Bi, Liwei Pang, Tengqi Ren, Ye Yang, Yang Sun, Zhuyuan Zheng, Shuodong Wu, Jing Kong

**Affiliations:** ^1^ Biliary Surgery (2nd General) Unit, Department of General Surgery, Shengjing Hospital of China Medical University, Shenyang, China; ^2^ Innovation Institute of China Medical University, Shenyang, China; ^3^ Breast Surgery Unit, Department of General Surgery, The Second Affiliated Hospital Zhejiang University School of Medicine, Hangzhou, China; ^4^ Department of Urinary Surgery, Taizhou Enze Medical Center (Group) Enze Hospital, Taizhou, China

**Keywords:** osthole, anticancer, proliferation, apoptosis, metastasis, angiogenesis, chemotherapy, multidrug resistance

## Abstract

With its high incidence and mortality rates, cancer is one of the largest health problems worldwide. Investigating various cancer treatment options has been the focus of many domestic and international researchers, and significant progress has been made in the study of the anticancer effects of traditional Chinese medicines. Osthole, a coumarin compound extracted from *Cnidium monnieri (L.) Cuss*., has become a new research hotspot. There have been many reports on its anticancer effects, and recent studies have elucidated that its underlying mechanism of action mainly involves inhibiting cancer cell proliferation, inducing cancer cell apoptosis, inhibiting invasion and migration of cancer cells, inhibiting cancer angiogenesis, increasing sensitivity to chemotherapy drugs, and reversing multidrug resistance of cancer cells. This mini-review summarizes the research progress on the anticancer effects of osthole in recent years.

## Introduction

With the aging of the world population, the global cancer incidence has increased every year (Bagaev et al., 2021), and despite various clinical treatment options—including surgical treatment (Harter et al., 2021), radiotherapy (Leboulleux et al., 2022), chemotherapy (van Stein et al., 2021), endocrine therapy (Du et al., 2022), and targeted gene therapy (Qiu et al., 2021)—mortality rates remain high and prognosis remains poor, rendering the effects of these treatments barely satisfying (Force et al., 2021). Traditional Chinese medicines have been shown to have an important role in inhibiting cancer cell growth and metastasis or reducing chemotherapy adverse reactions while offering many advantages, including a wide variety of sources, low price, and good human tolerance (Lyu et al., 2021).

Osthole is the main active ingredient of the Chinese traditional herbal medicine *Cnidium monnieri (L.) Cuss.* (molecular formula: C_15_H_16_O_3_, molecular weight: 244.29; Li M. O. et al., 2021). In recent years, many studies have reported a wide range of pharmacological activities of osthole, including anticancer, anti-inflammatory, antioxidant, antipruritic, antiasthma, anti-osteoporosis, antibacterial and antiviral, immune regulatory, and fracture healing promoting (Jarzab et al., 2017; Sun et al., 2021). In addition, it is characterized by rare toxic side effects and a broad safety range, which ensures glorious development prospects (Hassanein et al., 2020).

A recent review has provided a detailed summary of articles published before 2019 studying the anticancer mechanisms of osthole (Ashrafizadeh et al., 2020). However, related studies continue to emerge as this topic remains in the hotspot of cancer research. This mini-review summarizes recent research published after 2019 with the aim to complement the abovementioned review of Ashrafizadeh et al. and provide a complete reference on the anticancer capabilities and mechanisms of osthole.

## Anticancer mechanism of osthole in various organ systems

**FIGURE 1:**
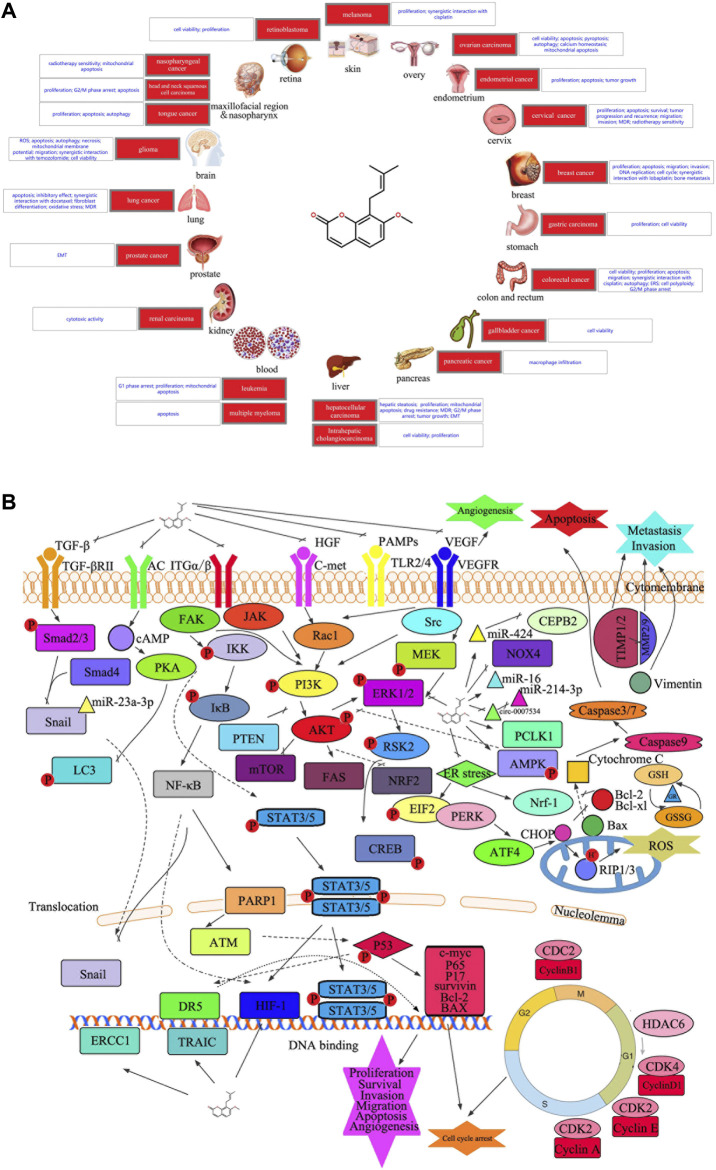
**(A)** Diseases involved in the current study of the anticancer potential of osthole. **(B)** Graphical summary of the anticancer mechanisms underlying the anticancer effect of osthole. Osthole acts on multiple signaling pathways in cancer cells to modulate several changes in phenotype, such as cell proliferation, apoptosis, cell cycle arrest, survival, migration, invasion, and angiogenesis. PKA, protein kinase A; AC, adenylate cyclase 1; cAMP, adenosine 3′,5′-cyclic phosphate; LC3, microtubule-associated protein 1 light chain 3 alpha; TGFBR2, TGF-beta receptor type-2; SMAD, mothers against decapentaplegic homolog; NF-κB, nuclear factor kappa B; IKK, I-kappa B kinase; IκB, I kappa B protein; PARP1, poly(ADP-ribose) polymerase 1; ATM, ATM serine/threonine kinase; DR5, tumor necrosis factor receptor superfamily, member 10b; ERCC1, ERCC excision repair 1; BID, BH3 interacting domain death agonist; ITGA/B, integrin alpha/beta; JAK, Janus kinase; RAC1, Ras-related C3 botulinum toxin substrate 1; FAK, focal adhesion kinase 1; STAT, signal transducer and activator of transcription; AMPK, 5′-AMP-activated protein kinase; PTEN, phosphatase and tensin; AKT, protein kinase B; PI3K, phosphatidylinositol 3-kinase; ERK, extracellular signal-regulated kinase; VEGF, vascular endothelial growth factor; MMP, matrix metalloproteinase; TIMP, tissue inhibitors of metalloproteinase; GSH, glutathione; GR, glutathione reductase; Bax, Bcl2-associated X protein; CHOP, trichoplein keratin filament-binding protein; GSSG, oxidized glutathione; MEK, mitogen-activated protein kinase; Nrf-1, nuclear respiratory factor 1; mTOR, mechanistic target of rapamycin kinase; CREB, cAMP-response element-binding protein; ROS, reactive oxygen species; FAS, fatty acid synthase; TLR, toll-like receptor; VEGFR, kinase insert domain protein receptor; PAMP, pathogen-associated molecular patterns; c-MET, proto-oncogene tyrosine-protein kinase Met; HGF, hepatocyte growth factor; miR, microRNA; c-myc, transcriptional regulator Myc-like; hif-1, hypoxia-inducible factor 1; P, phosphorylation; CDK, cyclin dependent kinases; NOX4, NADPH oxidase 4; CEPB, nonribosomal peptide synthetase CepB; HDAC, histone deacetylase; Nrf2, nuclear factor erythroid 2-related factor 2; TRAIL, tumor necrosis factor-related apoptosis-inducing ligand; EMT, epithelial-to-mesenchymal transition.

Osthole has broad-spectrum anticancer activities against multiple organ tumors (Ashrafizadeh et al., 2020). Advancements in cancer molecular biology research and development of new technology has enabled researchers to elucidate the mechanism of anticancer activity of osthole (Zafar et al., 2021). This activity tends to target numerous processes in the development of tumors (Figure 1A), and the molecular basis of targeting tumor cells is also very diverse (Figure 1B and Table 1).

**TABLE 1:** Anticancer effects of osthole against various organ cancers and side effects against normal organs.

Study number	Research target	Molecular target	Mode of action	References
Brain and nervous system				
1	Glioma cell lines (U87, C6)	RIP1, RIP3, mixed lineage kinase domain-like protein, caspase-3, caspase-7, caspase-9	ROS, apoptosis	Huangfu et al. (2021)
2	Glioblastoma multiforme (T98G) and anaplastic astrocytoma (MOGGCCM) cell lines	Beclin-1, PI3K, RAF, caspase-3	Apoptosis, autophagy, necrosis	Sumorek-Wiadro et al. (2020b)
3	Anaplastic astrocytoma (AA), glioblastoma multiforme (GBM) cell lines	Caspase-3, Bcl-2, beclin-1	Mitochondrial membrane potential, apoptosis, autophagy, migration, synergistic interaction with temozolomide	Sumorek-Wiadro et al. (2020a)
Respiratory system				
4	Lung cancer cell line (A549)	HDACs, caspase-9	Apoptosis	Abosharaf et al. (2020)
5	Lung adenocarcinoma cell line (A549)	No mention	Inhibitory effect, synergistic interaction with docetaxel	Jo et al. (2020)
6	BLM-induced pulmonary fibrosis in mice	NOX4	Pulmonary fibrosis, fibroblast differentiation, oxidative stress	Fang et al. (2021)
Digestive system				
7	HCC mouse model featuring hepatic steatosis transfected with AKT and c-Met, HCC cell line (HepG2, SMMC-7721)	Akt, p-AKT (Thr308), c-met, RPS6, FASN, PCNA, Ki67, ERK	Hepatic steatosis, proliferation	Mo et al. (2020)
8	CD133^+^ HCC cell line (HepG2, Huh7)	PTEN, AKT, p-AKT, Bcl-2, Bad	Mitochondrial apoptosis, drug resistance	Ye et al. (2020)
9	Hypoxic colon cancer cell line (HCT116)	EIF2α, ATF4, CHOP, DR5, HIF-1, cleaved caspase-3	Cell viability, proliferation, apoptosis, migration, synergistic interaction with cisplatin	Peng and Chou, (2022)
10	Colorectal cancer cell line (HT-29)	p62, LC3-II, LC3-I, GRP78, PERK, EIF2α, CHOP	Proliferation, autophagy, ERS, apoptosis	Zhou et al. (2021)
Reproductive system				
11	Highly metastatic breast cancer cell line (MDA-MB-231BO)	ITGα3, ITGβ5, FAK, Src, Rac1	Migration and invasion	Chen et al. (2021)
12	Breast cancer cell line (MDA-MB-231, MCF-7)	GNG7	Proliferation, apoptosis	Mei et al. (2021)
13	Triple-negative breast cancer cell line (MDA-MB-231)	p53, Bax, Bcl-2, caspase-3, p17	Proliferation, apoptosis, DNA replication, cell cycle, synergistic interaction with lobaplatin	Liu et al. (2021)
14	Cervical cancer cell line (HeLa, Me-180)	DCLK1	Cell proliferation, apoptosis, survival, tumor progression and recurrence	Pan et al. (2021)
15	Cervical cancer cell line (HeLa)	Wnt, *β*-catenin, c-Myc, cyclin D1, survivin, MMP-9	Proliferation, migration, invasion	Yin et al. (2021)
16	Ovarian cancer cell line (A2780, OVCAR3)	c-GSDME, LC3II	Cell viability, apoptosis, pyroptosis, and autophagy	Liang et al. (2020)
17	Ovarian cancer cell line (ES2, OV90)	PI3K, MAPK	Calcium homeostasis, mitochondrial apoptosis	Bae et al. (2021)
18	Endometrial cancer cell line (JEC), nude mouse xenograft model	PI3K, AKT, Bax, cleaved caspase-3, caspase-9, PARP	Proliferation, apoptosis, tumor growth	Liang et al. (2021)
19	Endometrial carcinoma cell line (Ishikawa, KLE)	CEPB2, miR-424	Proliferation, apoptosis	Lu et al. (2020)
Urinary system				
20	Renal cancer cell line (A498, UO31)	No mention	Cytotoxic activity	Tosun et al. (2020)
Blood and immune system				
21	Leukemia cell line (THP-1)	Mitochondrial membrane potential (ΛΨm)	G1 phase arrest, proliferation, mitochondrial apoptosis	Farooq et al. (2019)
Other organs				
22	HNSCC cell line (FaDu)	Bcl-2, ParP1, survivin, cyclin B1, CDC2, cleaved caspase-3/9, cleaved PARP1, Bax, PI3K, AKT	Proliferation, G2/M phase arrest, apoptosis	Yang et al. (2020)
23	Tongue cancer cell line (Tca8113)	LC3II, LC3I, P62, Bax, Bcl-2, cleaved caspase-3	Proliferation, apoptosis, autophagy	Sun et al. (2021)
24	Melanoma cell line (FM55P, FM55M2)	No mention	Proliferation, synergistic interaction with cisplatin	Wróblewska-Łuczka et al. (2021)
25	Retinoblastoma cell line (Y-79), nude mice injected with Y-79 cells	circ_0007534, miR-214-3p, PI3K, AKT, mTOR	Cell viability, proliferation	Lv et al. (2022)
Combination with chemotherapy drugs				
26	CD133^+^ HCC cell line (HepG2, Huh7)	PTEN, AKT, p-AKT, Bcl-2, Bad	MDR with cisplatin	Ye et al. (2020)
27	Lung adenocarcinoma cell line (A549)	No mention	Synergistic interaction with docetaxel	Jo et al. (2020)
28	Triple-negative breast cancer cell line (MDA-MB-231)	p53, Bax, Bcl-2, caspase-3, p17	Synergistic interaction with lobaplatin	Liu et al. (2021)
29	Melanoma cell line (FM55P, FM55M2)	No mention	Synergistic interaction with cisplatin	Wróblewska-Łuczka et al. (2021)
30	Anaplastic astrocytoma (AA), glioblastoma multiforme cell line (GBM)	Caspase-3, Bcl-2, beclin-1	Synergistic interaction with temozolomide	Sumorek-Wiadro et a., (2020b)
Side effects against normal organs				
31	SV40 transformed normal breast epithelial cell line (fR-2)	No mention	Breast epithelial cell toxicity	Farooq et al. (2019)
32	LAD2 mast cells	MRGPRX2, MRGPRB2, Ca^2+^ mobilization	Mast cell response	Callahan et al. (2020)
33	IL-4-induced rat alveolar macrophages line (NR8383)	NF-ĸB, MIF	Macrophage activation	Li M. O. et al. (2021)
34	Normal liver cell line (L-02)	Bcl-2, Bax, cleaved caspase-9/-8/-3, pro-caspase-3/-8, GRP78, Bip, CHOP, caspase-4, IRE1α, PERK, JNK, p-JNK, ATF4, p-histone H3, p-Cdc25C, Cdc25C, p-Cdc2, Cdc2, cyclin B1	Liver cell apoptosis	Shen et al. (2019)
35	Renal proximal tubular cell line (HK-2)	Klotho, JAK2, STAT1, STAT3	Renal tubular hypertrophy	Kan et al. (2019)
36	Astrocytes prepared from cerebral cortex of CCI mice	P2Y1R, p-JNK, mEPSPs, eEPSPs, GluA1, GluN2B, p-ERK, p-CREB, c-Fos	Astrocyte activation, inflammatory factor expression	Li R. et al. (2020)
37	Kainic acid--activated microglial cell line (BV-2)	Notch	Microglia activation and proliferation	Li Y. Z. et al. (2020)
38	Mice with ovariectomy (OVX)	Synaptic proteins, ERα, BDNF, TrKB, p-CREB, p-Akt, Rac1	*Hippocampus* protection	Adu-Nti et al. (2021)

ATF4, activating transcription factor 4; BDNF, brain-derived neurotrophic factor; BLM, bleomycin; c-GSDME, cleavage of gasdermin E; CDC2, cell division control protein 2; CEPB2, cytoplasmic polyadenylation element-binding protein 2; CHOP, C/EBP, homologous protein; CPEB2, cytoplasmic polyadenylation element-binding protein 2; DCLK1, doublecortin-like kinase protein 1; DR5, death receptor 5; EIF2α, eukaryotic initiation factor 2 alpha; ERK, extracellular signal-regulated kinase; ERS, endoplasmic reticulum stress; FAK, focal adhesion kinase; FASN, fatty acid synthase; GNG7, G protein gamma subunit 7; HIF-1, hypoxia-inducible factor 1; HDAC, histone deacetylase; HNSCC, head and neck squamous cell cancer; IRE1α, inositol-requiring transmembrane kinase/endoribonuclease 1α; ITG, integrin; JNK, c-Jun N-terminal kinase; LC3, microtubule-associated protein light chain 3; MAPK, mitogen-activated protein kinase; MDR, multidrug resistance; MIF, macrophage migration inhibitory factor; MMP-9, matrix metallopeptidase 9; MRGPR, mas-related G protein-coupled receptor; NF-ĸB, nuclear factor-kappa B; NOX4, NADPH, oxidase 4; PARP, poly adenosine diphosphate-ribose polymerase; PCNA, proliferating cell nuclear antigen; PERK, protein kinase R (PKR)-like endoplasmic reticulum kinase; PI3K, phosphoinositide 3-kinase; RAF, rapidly accelerated fibrosarcoma; RIP, receptor-interacting protein; ROS, reactive oxygen species; RPS6, ribosomal protein S6; TrKB, tropomyosin receptor kinase B; wnt, wingless-related integration site.

### Brain and nervous system

Human brain gliomas are the most common primary brain cancers in adults, with glioblastoma having the highest malignity; the medium survival of patients with glioblastoma is less than 16 months after surgery (Bagley et al., 2022). In a recent study, Huangfu et al. (2021) demonstrated that osthole induced necroptosis of glioma cell lines (U87 and C6) *via* reactive oxygen species production targeting the necroptosis protein receptor-interacting protein 1 (RIP1). The RIP1 inhibitor necrostatin-1 attenuated both osthole-induced necroptosis and the production of reactive oxygen species. Osthole had no effect on HEB cells until its concentration increased to 640 μM, but the LDH release treated with Osthole in glioma cells also increased significantly with 200 μM osthole (Huangfu et al., 2021).

In glioblastoma multiforme (T98G) and anaplastic astrocytoma (MOGGCCM) cell lines, osthole treatment at a concentration of 200 µM with an isoprenyl moiety as the most effective compound among simple coumarins was reported to regulate cell apoptosis, autophagy, and necrosis. Sorafenib reduced the levels of autophagy beclin-1, and phosphoinositide 3-kinase (PI3K), and rapidly accelerated fibrosarcoma kinase separately were proved to be the regulation target. As controls, T98G and MOGGCCM cells were incubated only with 0.01% of dimethyl sulfoxide (DMSO; Sumorek-Wiadro et al., 2020a). In their subsequent study, osthole correlated with the formation of Bcl-2/Beclin-1 complex, eliminating apoptosis in anaplastic astrocytoma and glioblastoma multiforme cell lines, as well as displayed an antimigratory potential through co-incubation with temozolomide. The osthole concentration of 150 μM was the most efficient and initiated apoptosis in approx. After the incubation with 200 μM of osthole, the number of apoptotic cells did not change. Necrosis dominated in T98G after the treatment with 250 μM of osthole (Sumorek-Wiadro et al., 2020b).

### Respiratory system

Lung cancer is currently the most common pulmonary malignancy (Tan and Tan, 2022). With the development of society, lung adenocarcinoma has become the most common histopathological type of lung cancer, accounting for about 50% of non-small-cell lung cancer cases (Myers and Wallen, 2022). Abosharaf et al. (2020) reported that osthole concentrations with IC_50_ 188.5 μM competitively inhibited histone deacetylase to induce apoptosis in a lung cancer cell line (A549) when compared with cisplatin as a positive control. Jo et al. (2020) found that docetaxel (DTX) and osthole at an optimal ratio of 1:4 were encapsulated in methoxy poly(ethylene glycol)-*b*-poly(caprolactone) (mPEG-*b*-PCL) micelles in a lung adenocarcinoma cell line (A549). In both *in vitro* and *in vivo* studies, DTX/osthole-loaded mPEG-*b*-PCL micelles showed a higher inhibitory effect than the single solution in A549 cells. The IC_50_ value of the DTX/OTH free drug was 1,219 nM and that of DTX/OTH-loaded mPEG-b-PCL was 2852 nM. (Jo et al., 2020).

Multicenter large-sample studies in the United Kingdom and the United States have confirmed that several occupational and environmental exposure factors could increase the risk of lung cancer in patients with idiopathic pulmonary fibrosis (Hubbard et al., 1996). Osthole significantly attenuated bleomycin-induced pulmonary fibrosis in mice by downregulating TGF-*β*1/NADPH oxidase 4-mediated oxidative stress in lung fibroblasts (Fang et al., 2021).

### Digestive system

According to the World Health Organization 2020 report, hepatocellular cancer (HCC) is the sixth most common type of cancer and the third most common cause of cancer-related mortality (Sung et al., 2021). In an HCC mouse model with hepatic steatosis combined with an HCC cell line (HepG2 and SMMC-7721), osthole (244 mg/kg) exerted HCC antilipogenic and antiproliferative activities by suppressing the phospho-protein kinase B (AKT) (Thr308)/ribosomal protein S6/fatty acid synthase axis and extracellular signal-regulated kinase phosphorylation *in vivo* and *in vitro* (Mo et al., 2020). In a CD133^+^ HCC cell line (HepG2 and Huh7), osthole reestablished sensitivity to cisplatin treatment with mitochondrial apoptosis expanding *via* the PTEN/AKT pathway. In addition, osthole (10 μmol/L) decreased the IC_50_ of cisplatin to CD133^-^ cells (Ye et al., 2020).

Colorectal cancer can be regarded as a marker of economic development. The increase in incidence among people of younger age is attributed to the diet model, increased body weight, lifestyle, and other factors (Siegel et al., 2020; Sung et al., 2021). Peng and Chou (2022) found that osthole (80 μM) significantly activated unfolded protein response signaling, particularly the phospho-eukaryotic initiation factor 2 alpha (EIF2α)/activating transcription factor 4/C/EBP homologous protein/death receptor 5 cascade proapoptotic pathway to attenuate cellular viability, proliferation, and migration in a hypoxic colon cancer cell line (HCT116). Furthermore, cotreatment of hypoxic HCT116 cells with osthole greatly increased the sensitivity to cisplatin (Peng and Chou, 2022). In another study, osthole (100, 50 and 25 µM) could significantly suppress cellular proliferation and viability in a colorectal cancer cell line (HT-29) treated with 0.1% DMSO as the control and induce cell apoptosis *via* autophagy and endoplasmic reticulum stress (Zhou et al., 2021).

### Reproductive system

Breast cancer has the highest incidence and mortality rate among women worldwide (Vranic et al., 2021). Currently, there is a lack of treatment-specific targets for triple-negative breast cancer, the most malignant form of breast cancer (Bianchini et al., 2022). In a highly metastatic breast cancer cell line (MDA-MB-231BO), osthole downregulated the expression of integrin α3 and integrin β5, which was upregulated in highly metastatic breast cancer, and attenuated cell migration and invasion possibly *via* suppression of focal adhesion kinase/Src/Rac1. Osthole with IC_50_ values of 24.2, 6.8, and 123.9 μg/ml (48 h) against MDA-MB-231, MDA-MB-231BO, and MCF-7 cells. Besides, osthole displayed a lower cytotoxicity against normal MCF-10A cells with an IC_50_ value of 8944.0 μg/ml (Chen et al., 2021). A recent study demonstrated that low G protein gamma subunit 7 expression in breast cancer tissues was associated with shorter overall and relapse-free survival and that osthole targeted G protein gamma subunit 7 to inhibit cell proliferation and promote apoptosis in breast cancer cell lines (MDA-MB-231, MCF-7; Mei et al., 2021). Liu et al. (2021) found that an osthole concentration of 50 μM decreased cell proliferation activity by 40%, inhibited the growth of a triple-negative breast cancer cell line (MDA-MB-231) in a concentration-dependent manner, and significantly inhibited cell proliferation. The enrichment analysis of transcriptome sequencing between the control and osthole treatment groups indicated that the differentially expressed genes play a role in apoptosis, p53 signaling, DNA replication, and cell cycle (Liu et al., 2021).

Cervical cancer is the most common of gynecological cancers (Castle et al., 2021). Osthole could competitively bind to doublecortin-like kinase protein 1 and interact with Val468 residues of doublecortin-like kinase protein 1 to form hydrogen bonds, affecting cellular proliferation and apoptosis, survival, cancer progression, and recurrence in cervical cancer cell lines (HeLa, Me-180). IC_50_ values of Osthole on HeLa and Me-180 were 45.01 ± 3.91 μM and 88.95 ± 0.13 μM, respectively, while values were 8.35 ± 1.62 μM and 10.29 ± 0.18 μM for LRRK, respectively (Pan et al., 2021). Osthole (50, 100, 200 μg/ml) extract also effectively promoted apoptosis and inhibited cellular proliferation, migration, and invasion potential in a cervical cancer cell line (HeLa) in a dose-dependent manner through inactivation of the Wnt/*β*-catenin pathway (Yin et al., 2021).

Ovarian cancer has the highest mortality rate among gynecological cancers, representing a serious threat to women’s health and life worldwide (Menon et al., 2018). A study on ovarian cancer cell lines (A2780 and OVCAR3) showed that osthole triggered pyroptosis in ovarian cancer cells through the cleavage of gasdermin E to induce LC3II-mediated autophagy, of which IC_50_ values were 73.58 and 75.24 μM in OVCAR3 and A2780 cells, respectively, with 5-Fu as a positive control (Liang et al., 2020). Osthole was found to target cancer cells, but not normal cells. As overall IC_50_ values for osthole in ovarian cancer cells were 20 μM through targeting the PI3K/mitogen-activated protein kinase signaling pathway to facilitate endoplasmic reticulum–mitochondrial axis-mediated anticancer mechanisms and exert calcium-dependent pharmacological potential in ovarian cancer cell lines (ES2, OV90; Bae et al., 2021).

In recent years, the incidence of endometrial cancer has been increasing (Crosbie et al., 2022). Osthole (200 µM) was found to inhibit cellular proliferation and induce apoptosis in an endometrial cancer cell line (JEC) by inhibiting the PI3K/AKT signaling pathway, and these findings were confirmed in an *in vivo* study of cancer cell growth in a nude mouse xenograft model (Liang et al., 2021). In another study, an increasing dose of osthole (50, 100, 200 μM) inhibited cellular proliferation and induced apoptosis in endometrial cancer cell lines (Ishikawa, KLE) by upregulation of miR-424 and downregulation of its target gene cytoplasmic polyadenylation element-binding protein 2 (Lu et al., 2020).

### Urinary system

Renal cell cancer is the most invasive malignant tumor of the urinary system and accounts for 2–3% of malignant tumors in adults (Lee et al., 2021). Tosun et al. (2020) found that osthole exerted selective cytotoxic activities in a renal cell cancer cell line (A498, UO31).

### Blood and immune system

Acute leukemia is a blood malignancy that seriously threatens human health. Chemotherapy remains the most popular treatment method; however, treatment options are limited in patients with disease recurrence (Groll et al., 2021). Farooq et al. (2019) found that osthole displayed anticancer effects in a leukemia cell line (THP-1). In addition, the author synthesized a novel series of osthole compounds, and most of the compounds displayed a higher antiproliferative activity and mitochondrial apoptotic potential than the parent osthole in THP-1 cells.

### Other organs

Osthole has also been found to exert anticancer activity in cancers of other organs. In a study conducted by Yang et al. (2020), osthole exhibited suppressive effects in a head and neck squamous cell cancer cell line (FaDu) by inducing cell cycle arrest (G2/M phase) and apoptosis *via* the PI3K/AKT pathway. IC_50_ values measured in FaDu cells were 122.35 ± 11.63 and 93.36 ± 8.71 μM after treatment with osthole for 24 and 48 h, respectively. Sun and Liu (2021) showed that osthole (40, 80, 120, 160 μM/L) could inhibit cellular proliferation in a tongue cancer cell line (Tca8113) by promoting cellular apoptosis and blocking autophagy flow through increasing LC3II and P62 and reducing LC3I levels. Wróblewska-Łuczka et al. (2021) found that osthole had the highest anticancer activity among coumarins (osthole, xanthotoxin, xanthotoxol, isopimpinellin, and imperatorin) in melanoma cell lines (FM55P, FM55M2). In addition, osthole could combine with cisplatin in a synergistic interaction to affect melanoma cells. The IC_50_ values of osthole oscillate from approximately 75 μM for human ovarian cancer cells to 46.2 μM for lung cancer cells, 42.4 μM for breast cancer cells, 24.8 μM for prostate cancer cells, and 23.2 μM for the human squamous carcinoma cell line A431 (Wroblewska-Luczka et al., 2021). *In vivo* and *in vitro* experiments using a retinoblastoma cell line (Y-79) showed that osthole with an IC_50_ of 200 μM for 24 h treatment and 120 μM for 48 h treatment significantly altered the circ_0007534/miR-214-3p pathway, affecting the PI3K/AKT/mTOR pathway and reducing cell viability (Lv et al., 2022).

## Combination with chemotherapy drugs

Multidrug resistance can cause chemotherapy failure in many patients and delay the timing of suitable treatment (Hanssen et al., 2021). Osthole could partially reverse cisplatin resistance in CD133-positive HCC cells *in vitro* and *in vivo* (Ye et al., 2020). Many studies have also shown that osthole could increase the sensitivity to chemotherapy drugs. In the study of Jo et al. (2020), osthole exhibited a synergistic effect with DTX in the treatment of lung adenocarcinoma. Liu et al. (2021) found that osthole enhanced the apoptosis-mediated growth inhibitory effect of lobaplatin in breast cancer cells with obvious effects on the related proteins (p53, Bax, Bcl-2, and caspase-3 p17). The combination with cisplatin resulted in the most desirable synergistic interaction to affect melanoma cells (Wroblewska-Luczka et al., 2021). Combined with temozolomide, an oral chemotherapy drug used in the treatment of glioma (Karachi et al., 2018), osthole could promote and enhance antiglioma potential (Sumorek-Wiadro et al., 2020a).

## Effects on normal organs

Osthole exerts a certain effect on normal cells while targeting cancer cells. This action is one of the side effects that researchers should consider in the clinical transformation of osthole. In the study of Farooq et al. (2019), osthole was effective against leukemia and showed less toxicity against normal cells.

The intercellular communication in the tumor immune microenvironment provides an important niche for tumorigenesis and cancer progression (Li R. et al., 2021). While exhibiting its effects on cancer cells, osthole can also affect many types of immune cells in the tumor immune microenvironment. Activated mast cells release several regulators that promote angiogenesis and cancer growth (Afrin et al., 2021); however, Callahan et al. (2020) showed that osthole could inhibit MRGPRX2/MrgprB2 (mouse ortholog of human MRGPRX2)-induced mast cell responses. Activated macrophages selectively kill cancer cells but not normal cells (Cleveland et al., 1974). In the treatment of asthma, osthole could reduce interleukin 4-induced translocation of nuclear factor-kappa B in an alveolar macrophage cell line (NR8383) to ameliorate macrophage activation (Li S. et al., 2021).

Liver and kidney toxicity is also a key indicator for measuring the side effects of drugs. Studies examining the liver and kidney toxicity potential of osthole found that it exerted toxic effects in a normal liver cell line (L-02) by inducing apoptosis *via* inhibiting cell proliferation, arresting the cell cycle at the G2/M phase, and activating endoplasmic reticulum stress (Shen et al., 2019). Additionally, osthole attenuated renal tubular hypertrophy to a certain degree (Kan et al., 2019).

Furthermore, osthole affects normal cells of the nervous system. In the treatment of neuropathic pain, osthole could inhibit astrocyte activation and reduce inflammatory factor expression by inhibiting the P2Y_1_-receptor-dependent JNK signaling pathway (Li R. et al., 2020). In the treatment of epilepsy, osthole downregulated notch signaling components to trigger a proliferation change in kainic acid--activated microglial cells (BV-2) (Li Y. Z. et al., 2020). Osthole treatment was shown to improve cognitive deficit and alleviate anxiety- and depression-like behaviors induced by ovariectomy, the standard surgical treatment for ovarian cancer (Adu-Nti et al., 2021).

Therefore, the anticancer effects of osthole should be carefully weighed in consideration of its side effects, including those on the immune and nervous systems, as well as its liver and kidney toxicity.

## Conclusion and future perspectives

In summary, a growing body of evidence demonstrates the anticancer effects of osthole. Numerous studies have shown that osthole can significantly inhibit the growth of various cancer cells, and the underlying mechanisms of its effects included inhibition of cellular proliferation, induction of apoptosis, reversal of multidrug resistance, and inhibition of cancer cell invasion and migration. Furthermore, osthole could enhance the anticancer effect of some chemotherapy drugs. Based on the above results, we can suggest the factors underlying its anticancer activity. While the development of modern pharmacological experimental technology and diversification of research methods have promoted the investigation and discovery of the pharmacological effects of osthole, its clinical application in the treatment of cancer is relatively unexplored. For this purpose, researchers should consider investigating the therapeutic window against toxic effects. We believe that the advancement of the medical and pharmacology sciences will contribute to the wide use of osthole as stable anticancer drugs in clinical practice.
